# Metabolomic Analysis Reveals the Association of Severe Bronchopulmonary Dysplasia with Gut Microbiota and Oxidative Response in Extremely Preterm Infants

**DOI:** 10.3390/metabo14040219

**Published:** 2024-04-13

**Authors:** Chih-Yung Chiu, Ming-Chou Chiang, Meng-Han Chiang, Reyin Lien, Ren-Huei Fu, Kai-Hsiang Hsu, Shih-Ming Chu

**Affiliations:** 1 Division of Pediatric Pulmonology, Department of Pediatrics, Chang Gung Memorial Hospital at Linkou, and Chang Gung University, Taoyuan 333, Taiwan; 2 Clinical Metabolomics Core Laboratory, Chang Gung Memorial Hospital at Linkou, Taoyuan 333, Taiwan; neo0914@cgmh.org.tw; 3 Division of Pediatric Neonatology, Department of Pediatrics, Chang Gung Memorial Hospital at Linkou, and Chang Gung University, Taoyuan 333, Taiwan; cmc123@cgmh.org.tw (M.-C.C.); reyinl@cgmh.org.tw (R.L.); rkenny@cgmh.org.tw (R.-H.F.); khsu@cgmh.org.tw (K.-H.H.)

**Keywords:** antioxidative capacity, bronchopulmonary dysplasia, gut microbiota, urinary metabolomics, very preterm infants

## Abstract

Bronchopulmonary dysplasia (BPD) is a chronic lung disease mainly affecting premature infants needing ventilation or oxygen for respiratory distress. This study aimed to evaluate the molecular linkages for BPD in very and extremely preterm infants using a metabolomics-based approach. A case-control study of enrolling preterm infants born before 32 weeks gestational age (GA) was prospectively performed. These preterm infants were subsequently stratified into the following two groups for further analysis: no or mild BPD, and moderate or severe BPD based on the 2019 NICHD criteria. Urinary metabolomic profiling was performed using ^1^H-Nuclear magnetic resonance (NMR) spectroscopy coupled with partial least squares discriminant analysis (PLS-DA) at a corrected age of 6 months. Metabolites significantly differentially related to GA and BPD severity were performed between groups, and their roles in functional metabolic pathways were also assessed. A total of 89 preterm infants born before 32 weeks gestation and 50 infants born at term age (above 37 completed weeks’ gestation) served as controls and were enrolled into the study. There were 21 and 24 urinary metabolites identified to be significantly associated with GA and BPD severity, respectively (*p* < 0.05). Among them, N-phenylacetylglycine, hippurate, acetylsalicylate, gluconate, and indoxyl sulfate were five metabolites that were significantly higher, with the highest importance in both infants with GA < 28 weeks and those with moderate to severe BPD, whereas betaine and N,N-dimethylglycine were significantly lower (*p* < 0.05). Furthermore, ribose and a gluconate related pentose phosphate pathway were strongly associated with these infants (*p* < 0.01). In conclusion, urinary metabolomic analysis highlights the crucial role of gut microbiota dysbiosis in the pathogenesis of BPD in preterm infants, accompanied by metabolites related to diminished antioxidative capacity, prompting an aggressive antioxidation response in extremely preterm infants with severe BPD.

## 1. Introduction

The global incidence of preterm birth is estimated at 10.6% according to the World Health Organization (WHO) [1]. The preterm birth rate in Taiwan increased by 11.1% (from 8.2% in 2001 to 9.1% in 2011), which may be attributed to improvements in maternal healthcare, prenatal care, and neonatal medicine [2]. Clinically, preterm birth is frequently associated with a range of complications and increased morbidity, including respiratory issues, neurological problems, feeding difficulties, increased infection risks, cardiovascular challenges, and growth and developmental delays.

Preterm infants, especially very and extremely preterm (born before 32 and 28 weeks of gestation, respectively), are at a higher risk of developing BPD [3]. The inflammatory response triggered by prematurity, lung immaturity, and the need for respiratory support can lead to lung injury and impaired lung development [4]. This inflammation contributes to the pathogenesis of BPD, involving abnormal lung tissue growth, impaired airway development, and disruption of normal lung structure. However, the molecular mechanisms underlying the inflammatory processes involved in BPD have not been fully elucidated.

The symptoms of BPD gradually improve over time as the lungs continue to mature and heal. Despite there being a general improvement in lung function and a reduction in the symptoms associated with BPD within the first 6 months, most infants experience a significant reduction in symptoms by the age of 2 or 3 years [5,6]. Metabolomics employs nuclear magnetic resonance (NMR) spectroscopy to study distinct biochemical molecules and metabolic pathways in living systems due to NMR’s reproducibility and high-throughput molecular identification capabilities [7,8]. Urine, being a noninvasive biospecimen, is particularly advantageous for metabolomics research as it reflects metabolic breakdown products from daily intake, and abundant chemical composition have been identified by NMR [9]. This study was aimed to identify the metabolic signatures of BPD severity related to gestational age (GA) using ^1^H-NMR spectroscopy in the early life of preterm infants, hopefully providing a molecular mechanism for preventing and managing BPD through targeted interventions, thereby enhancing the long-term prognosis and well-being of affected infants.

## 2. Materials and Methods

### 2.1. Study Population

Preterm infants less than 32 weeks gestation and full-term infants greater than 37 weeks gestation, who were followed up regularly at the outpatient department, were prospectively enrolled into this study. Infants with congenital chromosome abnormalities, brain malformations, or airway anomalies were excluded. Demographic data including infant’s age, sex, body mass index (BMI), breastfeeding patterns, and comorbidities were recorded and analyzed. BPD was diagnosed as the requirement of oxygen supplementation either at 28 days postnatal age or 36 weeks postmenstrual age (PMA) based on the 2019 revision of National Institute of Child Health and Human Development (NICHD) criteria [3]. Grade 1, grade 2, and grade 3 BPD were, respectively, categorized as mild, moderate, and severe levels of BPD. All enrolled infants were stratified into the following three groups by gestational age: GA ≥ 37 weeks (full-term), GA 28–32 weeks (very preterm), and GA < 28 weeks (extremely preterm). They were also stratified into the following groups by BPD severity: healthy controls (HC), no or mild BPD (No + Mild BPD), and moderate or severe BPD (M + S BPD).

### 2.2. Urine Sample Preparation

The spot urine samples of the enrolled subjects were prepared following the previously described methodology [10]. In brief, a mixture of 100 μL of 1.5 M phosphate buffer in deuterium water, which included 0.04% 3-(trimethylsilyl)-propionic-2,2,3,3-d4 acid sodium salt (TSP), was combined with 900 μL of urine to serve as an internal chemical shift reference standard. The resulting mixture was subjected to vortexing for 20 s and centrifuged at 12,000× *g* at 4 °C for 30 min. Subsequently, 600 μL of the supernatant from the sample was transferred to a 5 mm NMR tube for further analysis.

### 2.3. ^1^H–Nuclear Magnetic Resonance (NMR) Spectroscopy

The ^1^H-NMR spectra were obtained using a Bruker Avance 600 MHz spectrometer (Bruker-Biospin GmbH, Karlsruhe, Germany) equipped with a 5 mm CPTCI ^1^H cryoprobe located at the Chang Gung Healthy Aging Research Center in Taiwan [10]. A total of 64 scans were collected, resulting in 64 K computer data points, with a spectral width of 10,000 Hz (10 ppm) and a relaxation time of 4 s for each spectrum. Preceding the zero-filled Fourier transformation, the 1D ^1^H-NMR spectra underwent processing, which included an exponential line broadening of 0.3 Hz. Subsequently, the obtained NMR spectra were subjected to manual phasing, baseline correction, and reference alignment to the chemical shift of TSP (δ 0.0 ppm) using TopSpin 3.2 software (Bruker BioSpin, Rheinstetten, Germany) [11].

### 2.4. NMR Data Processing and Analysis

The raw ^1^H-NMR spectra were imported into NMRProcFlow software [12]. To address misalignment among the NMR spectra, a combination of parametric time warping and least-squares algorithm was applied. The spectra were then subjected to metabolite analysis using the variable size and intelligent bucketing method. The identification of metabolites was performed using Chenomx NMR Suite 8.1 software (Chenomx Inc., Edmonton, AB, Canada). In order to account for differences in urinary concentration, the urine spectra were specifically normalized based on the integral of the creatinine peak at δ 3.045 ppm.

As with previous NMR data analysis methods [11], the normalized ^1^H-NMR spectra data underwent a generalized log transformation (glog) for further analysis. In order to identify discriminative metabolites between the groups, PLS-DA was conducted using MetaboAnalyst 5.0, an online tool [13]. The spectral variables were scaled and mean-centered using Pareto scaling. To evaluate the statistical models’ quality, diagnostic measures such as R^2^ and Q^2^ were calculated, employing a 10-fold internal cross-validation. Metabolites exhibiting a *p*-value < 0.05 between the groups were selected, and the functional metabolic pathways were analyzed using the Kyoto Encyclopedia of Genes and Genomes database (KEGG).

### 2.5. Statistical Analysis

Appropriate univariate nonparametric and parametric tests including the chi-square test, Fisher’s exact test, ANOVA, and the Kruskal–Wallis test by ranks were used to compare the baseline characteristics between children with different GA and BPD groups. The Mann–Whitney test was employed to assess the variations in metabolites between two groups, utilizing the MetaboAnalyst web server. VENNY 2.1 was used to create the Venn diagram (https://bioinfogp.cnb.csic.es/tools/venny/ (accessed on 3 January 2024)). The correlations between metabolites significantly differentially expressed in different GA and BPD severity were performed using Spearman’s correlation test in R software (Lucent Technologies, Murray Hill, NJ, USA, version 4.0.3). C-means clustering was used to group the significantly differential metabolites into discrete and stable clusters of time series data using the Mfuzz package. Metabolites in each cluster were analyzed and assessed through linear modelling, applying a single contrast between samples at different time points. Random forest models were employed for the independent ranking of metabolic profiles, and were validated using a 20-fold stratified cross-validation approach, incorporating the Boruta feature selection algorithm and classification [14]. Apart from this, the statistical analysis was conducted using the Statistical Package for the Social Sciences (SPSS) software, version 20.0 (SPSS Statistics for Mac, Armonk, NY, USA). A two-tailed *p*-value < 0.05 was deemed statistically significant.

## 3. Results

### 3.1. Population Characteristics

A total of 139 infants completed a 6-month follow-up period, of which 89 infants less than 32 weeks gestation were enrolled into this study. Among them, 48 infants were born between 28 and 32 weeks, and 41 infants were born before 28 weeks of pregnancy. There were 17 infants who were not diagnosed with BPD, while mild, moderate, and severe BPD were, respectively, diagnosed in 23, 41, and 8 infants. These preterm infants were subsequently stratified into the following two groups for further analysis: no or mild BPD (*n* = 40), and moderate or severe BPD (*n* = 49). The comparisons of the baseline characteristics between three groups categorized by different GA and BPD severity at the corrected age of 6 months are shown in Table 1. Compared to full-term infants, body weight and BMI were significantly lower in preterm infants with GA less than 28 weeks and with moderate to severe BPD (*p* < 0.01).

### 3.2. Urinary Metabolite Sets Categorized by Different GA and BPD Severity

^1^H-NMR spectra from urine samples corresponded to 44 known metabolites. There are 21 and 24 urinary metabolites between groups with different GA and BPD severity that have a *p*-value of <0.05 and are, respectively, shown in Table 2 and Table 3. A Venn diagram showed the distribution of the metabolites that associated with different GA and BPD severity (Figure S1A). Five common metabolites including N-phenylacetylglycine, acetylsalicylate, creatine, hippurate, and valine were significantly associated with GA and BPD severity. Furthermore, nine metabolites including betaine, N,N-dimethylglycine, gluconate, urea, maltose, 4-hydroxyphenylacetate, indoxyl sulfate, carnitine, and ribose were found to be involved in GA < 28 weeks and moderate or severe BPD, whereas four metabolites including dimethylamine, glutamine, 3-hydroxyisobutyrate, and pantothenate were unique to moderate or severe BPD. Among them, metabolites related to GA and BPD severity were strongly associated with gestational age and birth body weight in a widespread manner, but not body weight and BMI at the corrected age of 6 months (Figure S1B, *p* < 0.01). However, gluconate, glutamine, and dimethylamine related to moderate or severe BPD were significantly negatively correlated to their body weight and body height at 6 months of corrected age (*p* < 0.01).

### 3.3. Dynamic Metabolic Changes across Different GA and BPD Severity

**Figure 1:**
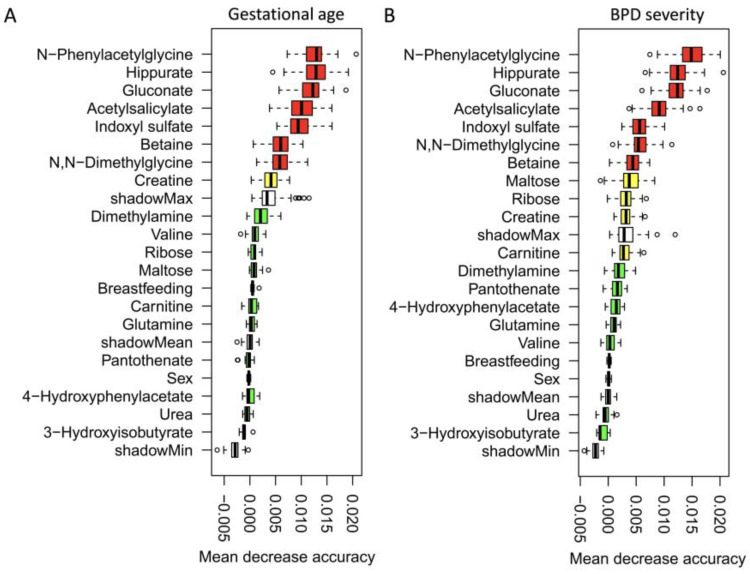
Markers for detecting children with different GA (**A**) and BPD severity (**B**) identified from random forest classifiers based on a combination of metabolites with baseline characteristics. Markers are ranked in descending order of their importance to the accuracy of the model. The boxes represent 25th–75th percentiles, and black lines indicate the median. GA, gestational age; BPD, bronchopulmonary dysplasia.

C-means clustering resulted in three modules of metabolites that showed distinctive temporal patterns of GA characterized by their initial abundance of metabolites and variation in expression at the different status of groups (Figure S2A). Compared to healthy infants, hippurate, N-phenylacetylglycine, 4-hydrophenylacetate, acetylsalicylate, ribose, creatine, and indoxyl sulfate were significantly higher in infants with GA < 28 weeks and with moderate to severe BPD (Figure S2B, *p* < 0.01). By contrast, betaine and N,N-dimethylglycine were significantly lower in these infants (*p* < 0.05). Furthermore, random forest regression models based on a combination of metabolites with baseline characteristics were performed to discriminate infants with different GA (Figure 1A) and BPD severity (Figure 1B). N-Phenylacetylglycine, hippurate, gluconate, acetylsalicylate, and indoxyl sulfate were confirmed to be the five metabolites with the highest importance for both GA and BPD severity.

### 3.4. Metabolic Pathway and Functional Analysis

**Figure 2:**
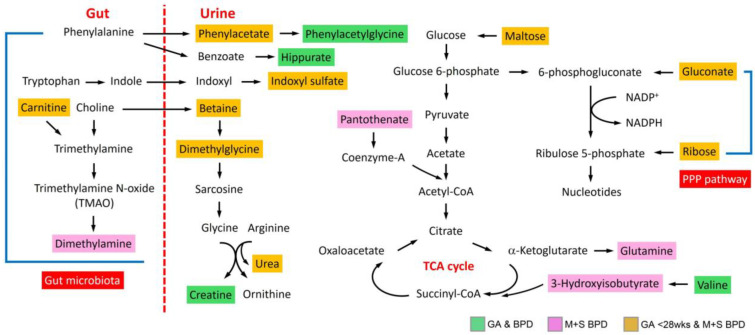
Schematic overview of the metabolites significantly associated with different GA and BPD severity and their related pathways. GA, gestational age; BPD, bronchopulmonary dysplasia; M + S BPD, moderate or severe BPD; TCA, tricarboxylic acid; PPP, pentose phosphate pathway.

The metabolic functional pathways related to metabolites that exhibit significant differences in GA and BPD groups, GA < 28 weeks, and moderate or severe BPD groups, as well as those unique to moderate to severe BPD groups selected by a *p*-value < 0.05 are shown in Table S1. Valine and hippurate related amino acid metabolisms were significantly associated with different GA and BPD severity (*p* < 0.05). However, ribose and gluconate related pentose phosphate pathway, and betaine and N,N-dimethylglycine related glycine, serine, and threonine metabolism were strongly associated with GA < 28 weeks and moderate or severe BPD groups (*p* < 0.01). Figure 2 presents a composite representation delineating significant metabolites alongside their potential functional pathways, elucidating the postulated molecular mechanisms.

## 4. Discussion

Preterm infants born before 32 weeks have a heightened risk of developing BPD due to inflammation caused by prematurity, lung immaturity, and respiratory support, leading to lung injury and disrupted lung development. BPD symptoms improve as infants’ lungs mature, with significant relief typically occurring within the first 6 months. However, the mechanisms underlying the molecular processes associated with the severity of BPD have not been fully explored. This study employs urine metabolomic analysis to elucidate the molecular linkages and pathophysiology of severe BPD development in very and extremely preterm infants.

The growth patterns and outcomes for preterm infants depend on various factors, including their gestational age at birth, overall health, and the quality of medical care they receive [15]. Preterm infants usually catch up in weight and may reach a size similar to that of full-term infants of the same age. However, in this study, extremely preterm infants, typically those born before 28 weeks of gestation, were found to be significantly smaller in size compared to full-term infants at 6 months of corrected age. It is essential to remember that extremely preterm infants have unique needs, and their developmental trajectory may differ significantly from that of full-term infants.

During growth, the diet provides adequate energy sources and protein, resulting in weight gain commensurate with age. Muscle mass is the major reservoir of protein in the human body, and glutamine is an important component of muscle protein, aiding in the repair and building of muscle [16]. In this study, a strong negative correlation was observed between urinary glutamine levels and body weight within the first half year of life in preterm infants. This correlation may be interpreted as a consequence of reduced skeletal muscle tissue growth during infancy, with the utilization of glutamine, particularly in cases of severe BPD.

N-Phenylacetylglycine, a product of phenylalanine breakdown, is influenced by the gut microbiota [17]. Hippuric acid, a host-microbe metabolite, primarily originates from the reductive metabolism of phenylalanine to yield benzoic acid in the gut, despite increased levels influenced by non-microbe pathways [18,19]. Premature birth disrupts the development of the neonatal gut microbiota, which comprises a community of microorganisms in the digestive system [20]. In this study, N-phenylacetylglycine and hippuric acid, linked to the gut microbiota, appeared to be the most important metabolites related to lower GA-associated severe BPD, emphasizing the role of gut microbiota dysbiosis in preterm infants on the pathogenesis of BPD severity.

Additionally, indoxyl sulfate, an indole derivative, could be produced from the amino acid tryptophan by gut microbiota to help maintain intestinal barrier integrity and immune cell homeostasis [21]. Preterm infants with severe BPD can experience implications for the development and alterations in the function of immune cells [22]. In this study, urinary indoxyl sulfate levels were found to be significantly higher in extremely preterm infants with severe BPD. These findings not only emphasize the significance of gut microbiota and dysbiosis in extremely preterm infants, but also highlight the crucial role of their immune system in infants with severe BPD.

Betaine and dimethylglycine are related compounds that have been investigated for health benefits, including their roles in cellular processes and their function as methyl donors in several metabolic pathways, such as DNA methylation, for potential antioxidant properties [23,24,25]. Oxidative stress plays a role in the development and progression of BPD [26]. Premature infants with underdeveloped lungs often require supplemental oxygen therapy, which is vital for their survival but can generate reactive oxygen species (ROS) in their lungs, potentially damaging lung tissue. In this study, both betaine and dimethylglycine were significantly lower in extremely preterm infants with severe BPD, supporting the idea that severe BPD in preterm infants is associated with elevated oxidative stress and decreased antioxidative ability [27].

The pentose phosphate pathway (PPP) plays a critical role in suppressing oxidative stress by maintaining cellular redox balance and providing essential precursors for nucleotide synthesis [28]. Dysregulation or deficiency of enzymes in this pathway can have significant health implications, leading to oxidative stress-related diseases and disorders. A significant increase in the ribose and gluconate-associated pentose phosphate pathway was observed in extremely preterm infants with severe BPD in this study, indicating the aggressive activity of antioxidation for maintaining cellular health and preventing various metabolic disorders in these infants.

The major limitations of this study include the relatively small sample size and the low sensitivity of the assay for low-abundance metabolites using ^1^H-NMR spectroscopy. Medical management during intensive care in hospitals, such as nutrition supplements, calcium gluconate, and steroid usage, may influence systemic metabolic profiles. However, an age-matched case-control design employed to collect subjects at the outpatient department has minimally eliminated this influence in this study. Despite these limitations, NMR has the advantage of providing reproducible and non-destructive measurements of various compounds. Most importantly, urine is a non-invasive sample that primarily reflects valuable insights into physiological status, dietary habits, and clinical conditions.

In conclusion, extremely preterm infants born before 28 weeks of gestation tend to exhibit significantly smaller size compared to full-term infants at 6 months of corrected age, emphasizing the importance of recognizing their distinct developmental needs and trajectories. N-Phenylacetylglycine and hippuric acid, both associated with gut microbiota, are identified as crucial metabolites linked to severe BPD in extremely preterm infants, highlighting the significant role of gut microbiota dysbiosis in the pathogenesis of BPD severity. The significantly reduced levels of betaine and dimethylglycine in extremely preterm infants with severe BPD suggest a strong association between severe BPD and diminished antioxidative capacity. Simultaneously, a significant increase in the ribose and gluconate-associated pentose phosphate pathway indicates aggressive antioxidation activity aimed at maintaining cellular health in such instances. However, further functional research is needed to comprehensively investigate these associations.

## Figures and Tables

**Table 1:** Comparisons of the demographic characteristics among full-term and preterm infants less than 32 weeks of gestational age categorized by different GA and BPD severity at the corrected age of 6 months.

	GA				BPD Severity			
Characteristics	≥37 Weeks (*n* = 50)	28–32 Weeks (*n* = 48)	<28 Weeks (*n* = 41)	*p*-Value	HC (*n* = 50)	No + Mild (*n* = 40)	M + S (*n* = 49)	*p*-Value
Sex, male	20 (40.0%)	22 (45.8%)	20 (48.8%)	0.688	20 (40.0%)	19 (47.5%)	23 (46.9)	0.714
Gestational age (wk)	39.1 ± 0.8	30.7 ± 0.9	26.1 ± 1.4	**<0.001**	39.1 ± 0.80	30.2 ± 1.70	27.2 ± 2.40	**<0.001**
Birth body weight (g)	3171.3 ± 365.7	1369 ± 273.9	775.8 ± 203.7	**<0.001**	3171.3 ± 365.7	1404.9 ± 287.2	843.4 ± 241.8	**<0.001**
Age, corrected (month)	6.89 ± 1.25	7.17 ± 3.03	7.83 ± 2.10	0.129	6.89 ± 1.25	7.62 ± 3.33	7.36 ± 1.95	0.293
Body weight (g)	7.87 ± 0.75	7.71 ± 1.24	7.14 ± 1.42	**0.009**	7.87 ± 0.75	8.05 ± 1.00	6.96 ± 1.40	**<0.001**
Body height (cm)	67.31 ± 2.63	66.36 ± 3.42	65.53 ± 5.05	0.081	67.31 ± 2.63	67.50 ± 3.15	64.74 ± 4.64	**<0.001**
BMI (kg/m^2^)	17.36 ± 1.25	17.44 ± 2.05	16.38 ± 1.55	**0.005**	17.36 ± 1.25	17.64 ± 1.60	16.39 ± 1.97	**0.001**
Breastfeeding ≥ 6 months	28 (56.0%)	15 (31.2%)	19 (46.3%)	**0.046**	28 (56.0%)	14 (35.0%)	20 (40.8%)	0.110

Data shown are mean ± SD or number (%) of patients as appropriate. GA, gestational age; BPD, bronchopulmonary dysplasia; HC, healthy controls; No + Mild BPD, no or mild BPD; M + S BPD, moderate or severe BPD; wk, week; g, gram; cm, centimeter; BMI, body mass index. ANOVA and the Kruskal–Wallis test were employed to analyze the continuous data with and without normal distribution, respectively. All *p*-values < 0.05, which are in bold, are significant.

**Table 2:** The VIP score and fold change of metabolites significantly differentially expressed between different gestational age groups.

		GA < 28 Wks vs. GA ≥ 37 Wks			GA 28–32 Wks vs. GA ≥ 37 Wks			GA < 28 Wks vs. GA 28–32 Wks		
Metabolites	Chemical Shift, ppm (Multiplicity)	VIP Score *	Fold Change †	*p* ‡	VIP Score	Fold Change	*p*	VIP Score	Fold Change	*p*
N-Phenylacetylglycine	7.404–7.448 (m)	2.17	1.73	**<0.001**	1.72	1.37	**0.005**	1.93	1.27	**0.019**
Creatine	3.928–3.941 (s)	2.16	1.43	**<0.001**	2.12	1.20	**0.023**	1.48	1.19	0.158
Acetylsalicylate	2.337–2.357 (s)	2.04	1.96	**<0.001**	2.16	1.57	**0.004**	1.13	1.25	0.266
Hippurate	7.529–7.580 (m)	1.89	1.55	**<0.001**	1.61	1.28	**0.006**	1.59	1.21	**0.039**
4-Hydroxyphenylacetate	6.855–6.883 (ddd)	1.75	1.66	**<0.001**	1.10	1.42	0.145	1.73	1.17	0.082
3-Methyl-2-oxovalerate	1.098–1.122 (d)	1.74	1.47	**<0.001**	1.34	1.28	**0.027**	1.54	1.15	**0.044**
Indoxyl sulfate	7.693–7.719 (d)	1.47	1.43	**<0.001**	0.72	1.12	0.232	1.81	1.27	**0.024**
1-Methylnicotinamide	9.245–9.320 (s)	1.23	1.41	**0.003**	1.47	1.38	**0.016**	0.29	1.02	0.726
Ribose	5.373–5.389 (d)	1.51	1.84	**0.004**	1.13	1.48	0.131	1.29	1.24	0.221
3-Hydroxy-3-methylglutarate	1.317–1.327 (s)	1.01	0.84	**0.005**	0.76	0.94	0.158	0.79	0.89	0.275
Betaine	3.260–3.274 (s)	1.40	0.79	**0.009**	0.94	0.90	0.222	1.32	0.87	0.205
Valine	1.045–1.057 (d)	0.91	1.16	**0.011**	1.54	1.43	**0.009**	0.51	0.81	0.490
N,N-Dimethylglycine	2.920–2.938 (s)	0.95	0.87	**0.018**	0.64	0.96	0.251	0.89	0.90	0.255
Urea	5.650–6.056 (s)	0.92	1.23	**0.023**	0.72	1.09	0.195	0.85	1.13	0.234
Carnitine	3.224–3.237 (s)	1.02	1.34	**0.030**	1.09	1.18	0.090	0.55	1.13	0.516
Maltose	5.402–5.410 (d)	0.86	1.30	**0.032**	0.70	1.08	0.148	0.82	1.20	0.266
Gluconate	4.643–4.670 (d)	0.94	2.11	**0.033**	0.23	1.02	0.603	1.51	2.08	0.060
Tyrosine	6.890–6.915 (ddd)	0.83	1.14	**0.035**	0.14	1.06	0.817	1.25	1.08	0.101
Trimethylamine N-oxide	3.274–3.282 (s)	0.76	1.17	**0.044**	1.13	1.16	**0.033**	0.01	1.01	0.991
Allantoin	5.391–5.402 (s)	0.46	1.17	0.327	1.75	1.38	**0.007**	1.26	0.85	0.154
Succinate	2.400–2.418 (s)	0.39	1.19	0.388	1.22	1.25	**0.045**	0.72	0.96	0.393

* VIP scores were obtained from PLS-DA and a VIP score > 1 was shown. † Fold changes were calculated by dividing the value of metabolites in the neonates with different gestational age groups. ‡ All *p* values < 0.05, which are in bold, are significant. VIP, Variable Importance in Projection; GA, gestational age; wks, weeks; multiplicity, m, multiplet; s, singlet; ddd, doublet of doublet of doublet; d, doublet.

**Table 3:** The VIP score and fold change of metabolites significantly differentially expressed between different severities of preterm infants with BPD and healthy controls.

		M + S BPD vs. HC			No + Mild BPD vs. HC			M + S BPD vs. No + Mild BPD		
Metabolites	Chemical Shift, ppm (Multiplicity)	VIP Score *	Fold Change †	*p* ‡	VIP Score	Fold Change	*p*	VIP Score	Fold Change	*p*
N-Phenylacetylglycine	7.404–7.448 (m)	2.14	1.74	**<0.001**	1.61	1.29	**0.019**	0.63	1.34	**0.008**
Acetylsalicylate	2.337–2.357 (s)	2.09	1.96	**<0.001**	2.08	1.49	**0.012**	1.29	1.31	0.143
Creatine	3.928–3.941 (s)	2.03	1.37	**<0.001**	2.35	1.22	**0.026**	0.77	1.12	0.340
Hippurate	7.529–7.580 (m)	1.87	1.57	**<0.001**	1.53	1.21	**0.019**	0.52	1.30	**0.019**
4-Hydroxyphenylacetate	6.855–6.883 (ddd)	1.84	1.77	**<0.001**	0.69	1.23	0.406	2.47	1.44	**0.012**
3-Methyl-2-oxovalerate	1.098–1.122 (d)	1.79	1.59	**<0.001**	1.04	1.09	0.104	2.15	1.45	**0.002**
1-Methylnicotinamide	9.245–9.320 (s)	1.46	1.49	**<0.001**	1.09	1.28	0.107	0.92	1.16	0.135
Indoxyl sulfate	7.693–7.719 (d)	1.40	1.40	**<0.001**	0.63	1.10	0.352	0.14	1.27	**0.026**
Valine	1.045–1.057 (d)	1.08	1.31	**0.004**	1.40	1.31	**0.027**	0.21	1.00	0.692
Dimethylamine	2.718–2.732 (s)	0.58	1.08	**0.008**	0.18	1.04	0.666	0.75	1.04	0.152
Ribose	5.373–5.389 (d)	1.37	1.83	**0.009**	1.26	1.43	0.123	0.75	1.28	0.324
Maltose	5.402–5.410 (d)	0.94	1.24	**0.010**	0.49	1.11	0.402	0.24	1.11	0.134
Trimethylamine N-oxide	3.274–3.282 (s)	0.91	1.20	**0.012**	0.94	1.13	0.115	0.07	1.06	0.407
Gluconate	4.643–4.670 (d)	0.97	1.95	**0.020**	0.08	0.99	0.878	0.18	1.97	**0.028**
Pantothenate	0.928–0.940 (d)	0.97	1.28	**0.021**	0.71	1.09	0.288	0.89	1.18	0.207
Glutamine	2.431–2.461 (dt)	0.80	1.22	**0.025**	0.10	1.01	0.861	1.47	1.21	**0.037**
Carnitine	3.224–3.237 (s)	1.03	1.37	**0.026**	1.06	1.12	0.128	0.35	1.22	0.375
Urea	5.650–6.056 (s)	0.86	1.20	**0.026**	0.77	1.10	0.219	0.87	1.09	0.330
Betaine	3.260–3.274 (s)	1.08	0.81	**0.031**	1.43	0.89	0.109	0.62	0.91	0.882
3-Hydroxyisobutyrate	1.064–1.09 (d)	0.86	1.39	**0.032**	0.36	1.05	0.546	0.82	1.33	0.122
N,N-Dimethylglycine	2.920–2.938 (s)	0.77	0.89	**0.042**	0.90	0.96	0.162	1.99	0.93	0.719
Allantoin	5.391–5.402 (s)	0.91	1.33	**0.048**	1.23	1.21	0.085	0.15	1.10	0.731
3-Hydroxy-3-methylglutarate	1.317–1.327 (s)	0.49	0.94	0.170	1.65	0.84	**0.005**	0.53	1.12	0.185
Propylene glycol	1.130–1.146 (d)	0.67	0.71	0.220	1.89	0.58	**0.033**	0.60	1.22	0.252

* VIP scores were obtained from PLS-DA and a VIP score > 1 was shown. † Fold changes were calculated by dividing the value of metabolites in the preterm infants with different severities of BPD by the healthy controls. ‡ All *p* values < 0.05, which are in bold, are significant. VIP, Variable Importance in Projection; BPD, bronchopulmonary dysplasia; M + S BPD, moderate or severe BPD; HC, healthy controls; No + Mild BPD, no or mild BPD; Multiplicity, s, singlet; m, multiplet; ddd, doublet of doublet of doublet; d, doublet.

## Data Availability

The datasets used and analyzed during this study are available from the corresponding author upon reasonable request. The data are not publicly available because of privacy restrictions.

## Supplementary Materials

The following supporting information can be downloaded at: https://www.mdpi.com/article/10.3390/metabo14040219/s1, Figure S1: Venn diagram of the distribution of the metabolites significantly associated with different GA and BPD severity, and heatmap of Spearman’s rank correlation coefficients between these metabolites and baseline characteristics; Figure S2: Dynamic metabolic changes across different GA, and heatmap of significances of differentially expressed metabolites in comparison with healthy infants; Table S1: Metabolic pathway and function analysis of metabolites clustered across different statuses of GA and BPD severity.
