# Supplementary material for: Metabolomic Analysis Reveals the Association of Severe Bronchopulmonary Dysplasia with Gut Microbiota and Oxidative Response in Extremely Preterm Infants
Source: Metabolites. 2024 Apr 13;14(4):219. doi: 10.3390/metabo14040219 (PMC11052141; doi:10.3390/metabo14040219)
Supplement: Supplementary file 1 [file metabolites-14-00219-s001.zip › metabolites-2943003-supplementary.pdf]

Supplementary Figures and Tables

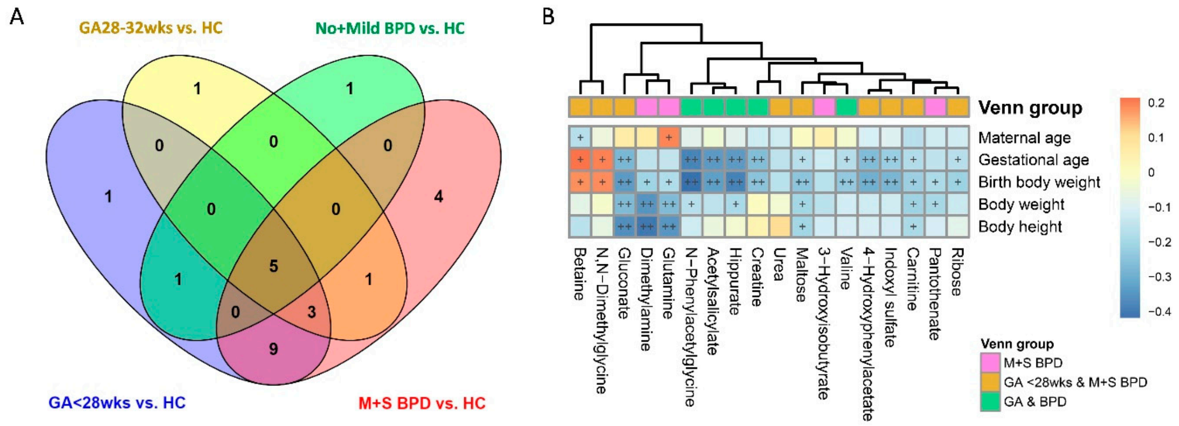

Figure S1. Venn diagram of the distribution of the metabolites significantly associated with different GA and BPD severity (A), and heatmap of Spearman's rank correlation coefficients between these metabolites and baseline characteristics (B). Total number of differentially expressed metabolites in each set and in the overlapping areas is indicated. Color intensity represents the magnitude of correlation. Red, positive correlations; blue, negative correlations. + symbol means a P-value < 0.05; ++ symbol means a P-value < 0.01. GA, gestational age; BPD, bronchopulmonary dysplasia.

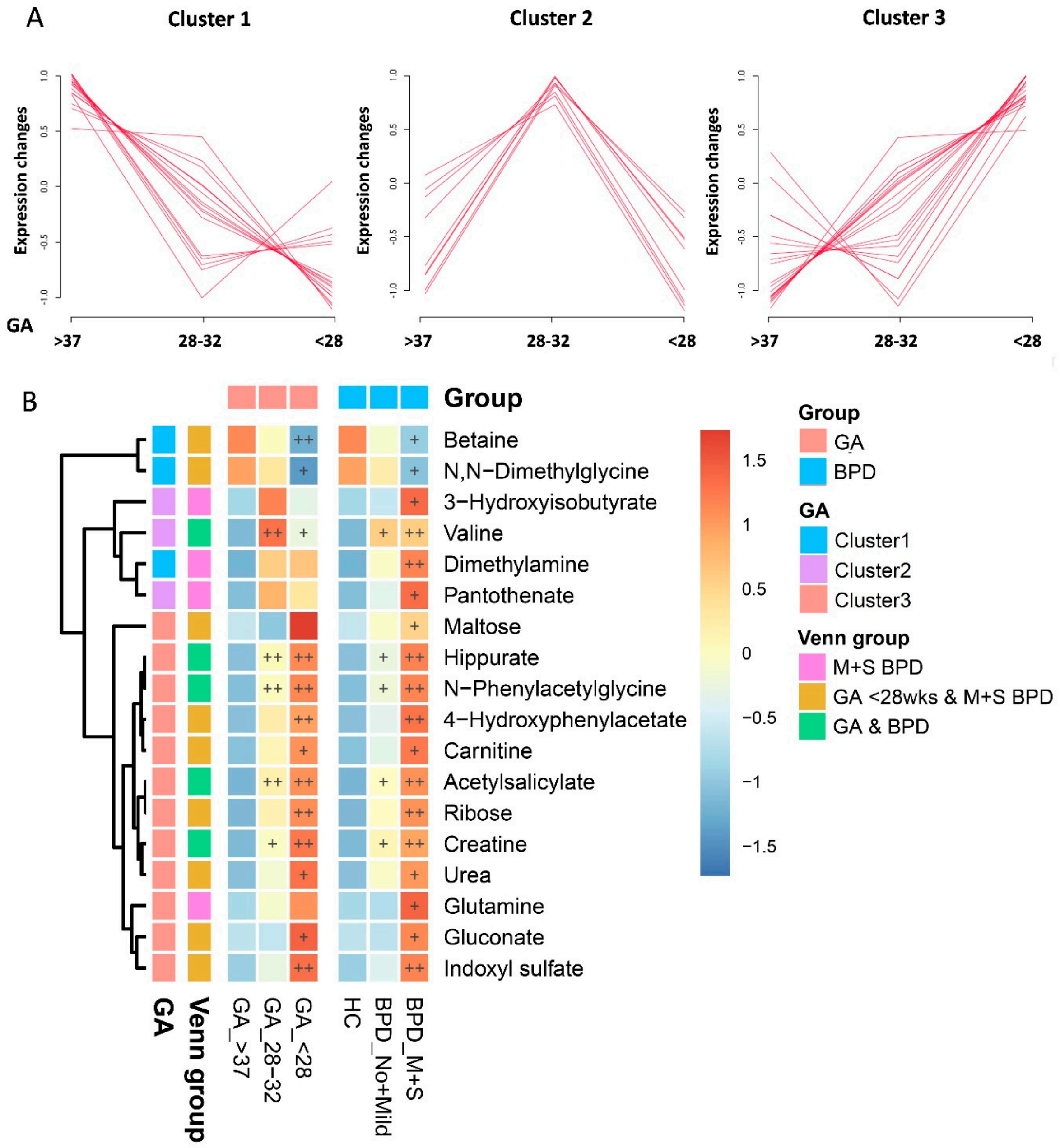

Figure S2. Dynamic metabolic changes across different GA (A), and heatmap of significances of differentially expressed metabolites in comparison with healthy infants (B). Color intensity represents the integral regions of NMR signals of metabolites. Red color represents increase in abundance, blue color represents relative decrease. + symbol means a *P*-value < 0.05; ++ symbol means a *P*-value < 0.01. GA, gestational age; BPD, bronchopulmonary dysplasia; NMR, nuclear magnetic resonance.

Table S1 Metabolic pathway and function analysis of metabolites clustered across different status of GA and BPD severity.

Metabolites	Pathway Name	Total	Hits	Raw*P*	FDR	Function
**GA & BPD**						
Valine	Valine, leucine and isoleucine biosynthesis	8	1	0.015	0.808	Amino acid metabolism
Hippurate	Phenylalanine metabolism	10	1	0.019	0.808	Amino acid metabolism
Valine	Pantothenate and CoA biosynthesis	19	1	0.036	1.000	Metabolism of cofactors and vitamins
**GA <28wks & M+S BPD**						
Ribose, Gluconate	Pentose phosphate pathway	22	2	0.003	0.234	Carbohydrate metabolism
Betaine, N,N-Dimethylglycine	Glycine, serine and threonine metabolism	33	2	0.006	0.263	Amino acid metabolism
**M+S BPD**						
Glutamine	D-Amino acid metabolism	6	1	0.012	0.486	Metabolism of other amino acids
Glutamine	Nitrogen metabolism	6	1	0.012	0.486	Energy metabolism

Total is the total number of compounds in the pathway; the Hits is the actually matched number from the user uploaded data; the Raw*P* is the original *P* value calculated from the enrichment analysis; the FDR is the portion of false positives above the user-specified score threshold. GA, gestational age; BPD, bronchopulmonary dysplasia; M+S BPD, moderate or severe BPD; FDR, false discovery rate.
